# The effects of arginase inhibitor on lung oxidative stress and inflammation caused by pneumoperitoneum in rats

**DOI:** 10.1186/s12871-015-0112-y

**Published:** 2015-09-28

**Authors:** Jin Sun Cho, Young Jun Oh, Ok Soo Kim, Sungwon Na

**Affiliations:** 1 Department of Anesthesiology and Pain Medicine, Yonsei University College of Medicine, Seoul, Republic of Korea; 2 Department of Anesthesiology and Pain Medicine, Anesthesia and Pain Research Institute, Yonsei University College of Medicine, 50 Yonsei-ro, Seodaemun-gu, Seoul 120-752 Republic of Korea; 3 Severance Biomedical Science Institute, Yonsei University College of Medicine, Seoul, Republic of Korea

**Keywords:** Arginase, Inflammation, Lung Injury, Nitric oxide, Oxidative stress, Pneumoperitoneum

## Abstract

**Background:**

Pneumoperitoneum-induced oxidative stress and organ injury are known to be associated with nitric oxide (NO) inactivation. Because arginase competes with NO synthase (NOS) for a common substrate, L-arginine, arginase inhibition may increase NO bioavailability. Therefore, we evaluated the ability of the arginase inhibitor, 2 (S)-amino-6-boronohexanoic acid (ABH), to attenuate pneumoperitoneum-induced decrease of NO bioavailability and lung injury.

**Methods:**

Thirty rats were randomly divided into the following groups: 1) the PP-ABH group received a subcutaneous injection of ABH (5 mg/kg) 1 h before induction of pneumoperitoneum (insufflation to intraperitoneal pressure of 15 mmHg for 60 min); 2) the PP group received saline by subcutaneous injection 1 h before induction of pneumoperitoneum; and 3) the control group received saline by subcutaneous injection before a sham procedure with no gas insufflation. After desufflation, blood was collected to determine levels of plasma nitrite, NOS, inflammatory cytokines, and malondialdehyde, a marker of oxidative stress. Lung tissue was obtained for histological evaluation.

**Results:**

We found that plasma nitrite levels were lower in the PP group and higher in the PP-ABH group, compared with controls (*P* <0.01 and *P* <0.05, respectively). In the PP group, endothelial NOS activity was decreased and inducible NOS activity was increased compared with the PP-ABH and control groups. Malondialdehyde levels increased 3-fold in the PP group and 2-fold in the PP-ABH group compared with controls. Tumor necrosis factor-α, interleukin-6, and interleukin-1ß levels were elevated in the PP group compared to the control group, but the increase in cytokine production was attenuated or blocked in the PP-ABH group. Lung injury scores were 4.8-fold higher in the PP group and 2-fold higher in the PP-ABH group compared with controls (*P* <0.001 and *P* <0.01, respectively).

**Discussion:**

Pneumoperitoneum decreases NO bioavailability and increases the inflammation cytokines, resulting in organ injuries. Inhibition of arginase activity could maintain NO bioavailability by attenuating pneumoperitoneum-induced changes in NOS activity. In addition, arginase inhibition attenuated the oxidative stress and inflammation and decreased the severity of lung injury caused by pneumoperitoneum.

**Conclusions:**

By increasing NO bioavailability and suppressing oxidative stress and inflammation, pretreatment with an arginase inhibitor may protect against lung injury caused by pneumoperitoneum.

## Background

Compared with conventional open surgery, minimally invasive surgery (e.g., laparoscopic and robotic surgery) has considerable advantages including less pain, shorter hospital stays, decreased morbidity, and more rapid return to full activity [1]. However, laparoscopic surgery is not without physiologic derangement of internal organs. Pneumoperitoneum required for laparoscopic visualization results in significantly decreased blood flow to intraperitoneal organs, which promotes anaerobic metabolism leading to lactic acidosis, oxidative stress, and postoperative manifestations, such as oliguria and elevated liver enzymes [2]. In addition, postoperative decrease in organ function is the major cause of laparoscopy-associated morbidity and mortality [3]. Even short periods of pressure as low as 10–15 mmHg cause organ hypoperfusion, leading to postoperative tissue injury [4–6]. More serious concerns have been raised about robot-assisted surgery, because it requires steep or reverse Trendelenburg positions, which are associated with higher risks of organ damage due to changes in intraperitoneal pressure [2].

Mechanisms of organ injury during pneumoperitoneum include decreased nitric oxide (NO), sympathetic hyperactivation, and oxidative stress [7–9]. Endothelial NO synthase (eNOS) produces most of the NO in the vasculature, where it activates soluble guanylyl cyclase and increases cyclic guanosine monophosphate levels in smooth muscle cells to dilate blood vessels [10]. In addition, NO inhibits platelet aggregation and suppresses vascular smooth muscle hyperplasia and its sensitivity to the sympathetic nerve activity [11, 12]. Therefore, decreased NO bioavailability directly causes vasoconstriction and indirectly inhibits vasodilation via the autonomic nervous system, resulting in increase in oxidative stress and ischemic damage of not only the intraperitoneal organ but the extraperitoneal organ as well [8].

Because eNOS competes with arginase for the substrate L-arginine, arginase inhibition has the potential to increase NO bioavailability [13]. The relationship between arginase inhibition and enhanced NO production has been demonstrated in animal studies using an arginase inhibitor or arginase knock-out model [14]. We reasoned that the deleterious effects of pneumoperitoneum on tissue perfusion could be attenuated by improving NO bioavailability. Specifically, we hypothesized that administration of an arginase inhibitor before pneumoperitoneum would increase NO bioavailability, thereby attenuating the pneumoperitoneum-induced lung injury. We tested our hypothesis by evaluating plasma nitrite, oxidative stress marker, inflammatory cytokines, and lung injury in a rat model during carbon dioxide (CO_2_) pneumoperitoneum with or without pretreatment with the arginase inhibitor 2 (S)-amino-6-boronohexanoic acid (ABH).

## Methods

### Animals and experimental design

This study was approved by the Yonsei University Institutional Animal Care and Use Committee and was conducted according to the established guiding principles for animal research. All experiments were performed in 3- to 4-month-old male Sprague–Dawley rats weighing 250–320 g. The rats were housed individually, maintained at a constant temperature and humidity under a 12-h dark/light cycle, and allowed food and water ad libitum. The rats were randomly divided into three groups, each consisting of 10 rats: 1) the PP-ABH group received a subcutaneous injection of ABH (5 mg/kg) 1 h before induction of pneumoperitoneum; 2) the PP group received a subcutaneous injection of saline 1 h before induction of pneumoperitoneum; and 3) the control group received saline only (pneumoperitoneum was not induced).

### Pneumoperitoneum

One hour after injection of ABH or saline, the rats were anesthetized with inhaled sevoflurane and secured a supine position on a thermostatically regulated heating pad (37 °C). After tracheostomy the lungs were mechanically ventilated with a volume-driven small-animal ventilator (model 665A, Harvard Apparatus, Holliston, MA, USA). Tidal volume was set 1 mL/100 g body weight, and respiratory rate was adjusted to 55–60 breaths/min to maintain end-tidal CO_2_ at 30–35 mmHg. Anesthesia was maintained with 1.5–2.0 % (volume percent) sevoflurane and 50 % oxygen in air. A Veress needle was then inserted in each rat. The control group underwent a sham procedure with no gas insufflation, maintaining intra-abdominal pressure at 0 mmHg. To induce pneumoperitoneum in the PP and PP-ABH groups, CO_2_ was insufflated using an electronic laparoflator (Karl Storz GmbH, Tuttlingen, Germany), maintaining intra-abdominal pressure at 15 mmHg. After 1 h of pneumoperitoneum or sham procedure, heparin was administered, and the rats were euthanized by cervical dislocation. Blood and lung tissue were obtained for analysis.

### Western blotting analysis for expression of NOS and arginase II

Lung tissue specimens were thawed and homogenized in ice-cold protein extraction buffer (Pro-prep, Intron Biotechnology, Seoul, Korea). The protein samples were separated by 8 %–10 % SDS-polyacrylamide gel electrophoresis, and then transferred to a 0.2-μm polyvinyl-difluoride membrane (Immun-Blot, Bio-Rad, CA, USA). The membranes were blocked using 5 % (wt/vol) nonfat milk in Tris-buffered saline with 0.1 % Tween-20, and then incubated overnight at 4 °C with polyclonal antibodies (1:1000 dilution) against inducible NOS (iNOS; Abcam®, UK), eNOS (Santa Cruz Biotechnology, CA, USA), and Arg II (Santa Cruz Biotechnology). The bound antibody was detected with labeled anti-rabbit secondary antibodies (1:5000 dilution) and visualized using the ECL Plus chemiluminescence assay (Amersham Biosciences, CA, USA). Protein expression was normalized to β-actin (1:1000 dilution).

### Arginase assay for arginase II activity

Activity of intracellular arginase was determined using the QuantiChrom™ arginase assay kit (BioAssay Systems, CA, USA), which measures the conversion of arginine to urea by arginase. Lung tissue was sonicated for 10 min in lysis buffer (50 mmol Tris–HCl, pH 7.5, 0.1 mmol EDTA, 0.1 % Triton X-100, and protease inhibitor) and centrifuged for 30 min at 14,000 × g at 4 °C. The supernatant (50 μL) was added to 75 μl Tris–HCl (50 nmol, pH 7.5) containing 10 mmol MnCl_2_, and the mixture was activated by heating at 55–60 °C for 10 min. The mixture was incubated with 50 μL L-arginine (0.5 M, pH 9.7) at 37 °C for 2 h, and the reaction was stopped by adding 400 μL acid solution (H_2_SO_4_:H_3_PO_4_:H_2_O, 1:3:7). For the colorimetric determination of urea, 25 μL α-isonitrosopropiophenone (9 % in ethanol) was added, and the mixture was heated at 100 °C for 45 min. After holding the reaction mixture in the dark for 10 min at room temperature, the urea concentration was determined spectrophotometrically by measuring absorbance at 430 nm.

### Griess nitrite assay

To measure plasma nitrite (NO_2_^−^), blood was collected in tubes containing anticoagulants (citrate and EDTA). The tubes were centrifuged for 15 min at 1000 × g, and the plasma was incubated with 100 μL Griess reagent (1 % sulfanilamide in 0.1 mol/l of HCl and 0.1 % N-(1-naphthyl) ethylene diamine dihydrochloride) (Sigma-Aldrich, St. Louis, MO, USA) at room temperature for 10 min. Absorbance at 540 nm was measured using a microplate reader, and nitrite content was calculated based on a standard curve constructed using NaNO_2_.

### Enzyme-linked immunosorbent assay for NOS activity

The activities of eNOS and iNOS were determined using rat eNOS and iNOS enzyme-linked immunosorbent assay (ELISA) kits (Yuhan EIAabscience, Korea). In brief, lung tissue specimens were homogenized in ice-cold Pro-Prep, and the supernatant was incubated in a 96-well plate containing co-factors and the substrate L-arginine at 37 °C. After 1 h, the reaction was quenched by adding the stop buffer. The concentrations of nitrites and nitrates in the reaction mixture were determined using a colorimetric method (530 nm).

### ELISA for oxidative stress marker malondialdehyde

To assess oxidative stress in the lung tissue, we evaluated malonyldialdehyde (MDA), which is a stable product of lipid peroxidation that is used as an index of oxygen free radical production. The MDA content in lung tissue was measured using an OxiSelect™ MDA Adduct ELISA kit (Cell Biolabs, Inc., CA, USA). After adding the stop solution to halt the enzymatic reaction, absorbance at 450 nm was measured using a spectrophotometer.

### ELISA for proinflammatory cytokines

Commercially available ELISA kits were used to determine serum levels of tumor necrosis factor (TNF)-α (BD Biosciences, CA, USA), interleukin (IL)-1β (eBioscience, CA, USA), and IL-6 (Young In Frontier Co., Ltd., Korea). In brief, supernatants of lung tissue were added to a 96 well plate coated with corresponding antibodies to each cytokine and incubated. Streptavidin-horseradish peroxidase conjugate was added as an antibody to a second epitope on each cytokine. Absorbance was read by a microplate reader using 450 nm as the primary wave length after adding the stop solution.

### Lung histology

Lung tissue specimens obtained from the left lower lobe were fixed with 3 % paraformaldehyde, embedded in paraffin, and then stained with hematoxylin and eosin. Lung injury was assessed by a pulmonologist, who was blind to the group allocation using the modified scoring system described by Kristof et al. [15]. The level of lung injury was graded as none (0), mild (1), moderate (2), or severe (3) for each of the following three categories: interstitial cellular infiltration, alveolar protein exudation, and tissue hemorrhage. For each rat, 10 microscopic fields were randomly selected (200× magnification). The score for each category was determined by adding the scores from the 10 different microscopic fields, and the total lung injury score for each rat was calculated as a sum of the three category scores.

### Statistical analysis

Results are expressed as mean ± standard error of the mean (SEM). Groups were compared by one-way analysis of variance for multiple comparisons (Tukey’s post hoc test). Nonparametric data were analyzed by chi-square test. Statistical analysis was conducted using Prism 5.01 software (GraphPad software Inc. CA, USA); *P* <0.05 was considered significant.

## Results

### ArginaseIIexpression and activity

**Fig. 1:**
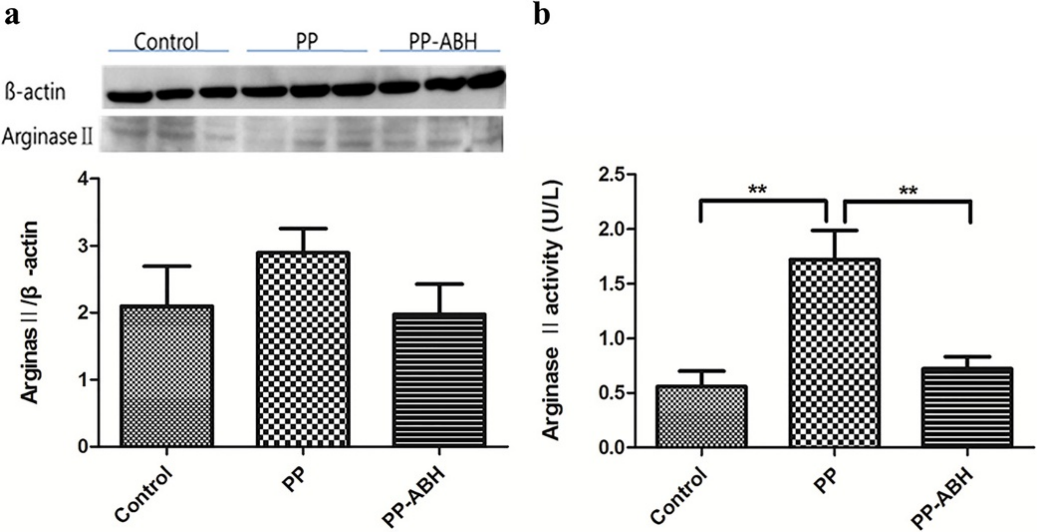
Arginase II expression and activity. **a** Arginase II expression did not differ significantly among the treatment groups. **b** However, arginase II activity was increased significantly in the PP group compared with the PP-ABH and control groups. ^**^P <0.01

Results of western blot analysis showed that arginase II expression in the lung tissue was comparable among the three groups of rats (Fig. 1a). However, arginase II activity was significantly higher in rats that underwent pneumoperitoneum (PP group), compared with those pretreated with ABH before pneumoperitoneum induction (PP-ABH group) and untreated controls (1.70 ± 0.60 U/L vs. 0.72 ± 0.25 U/L and 0.56 ± 0.31 U/L, respectively, *P* < 0.01 in both) (Fig. 1b).

### NO bioavailability

**Fig. 2:**
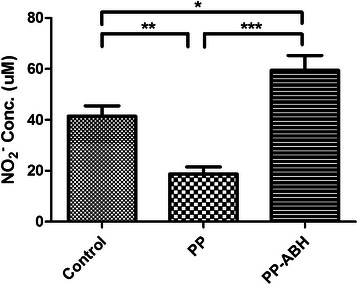
Plasma nitrite levels. Plasma nitrite, a stable metabolite of NO, was decreased in the PP group but increased in the PP-ABH group compared to controls. ^*^P <0.05, ^**^P <0.01, ^***^P <0.001

Level of plasma nitrite, a stable metabolite of NO, was significantly lower in the PP group compared with the PP-ABH and control groups (19 ± 6.1 μmol/L vs. 60 ± 1.3 μmol/L and 42 ± 9.0 μmol/L, respectively, *P* <0.001 and *P* <0.01, respectively) (Fig. 2). In addition, plasma nitrite level was higher in the PP-ABH group compared with the control group (*P* <0.05).

### NOS expression and activity

**Fig. 3:**
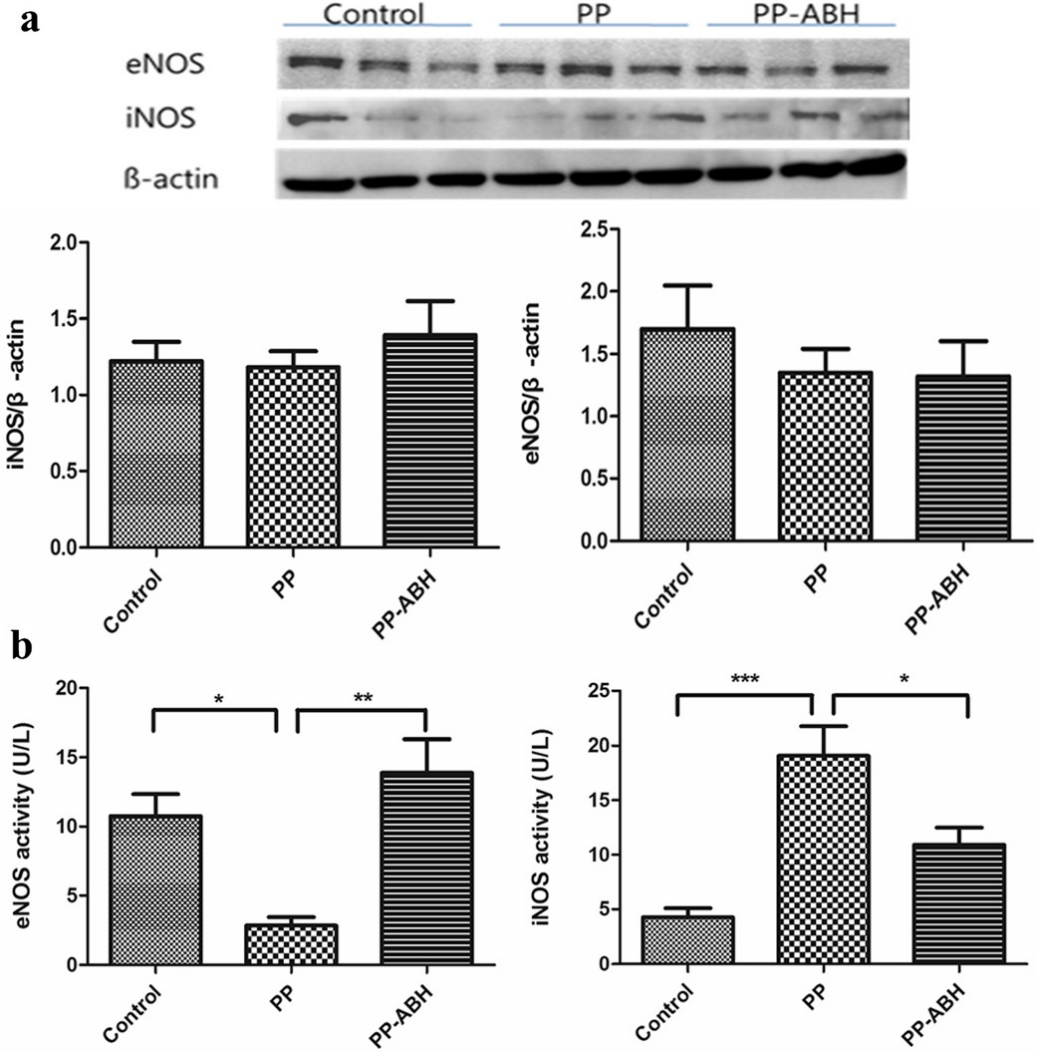
Expression and activity of endothelial and inducible nitric oxide synthase. **a** Expression of endothelial nitric oxide synthase (eNOS) and inducible nitric oxide synthase (iNOS) did not differ significantly among the three treatment groups. **b** However, eNOS activity was significantly lower and iNOS activity was higher in the PP group, compared with the PP-ABH and control groups. ^*^P <0.05, ^**^P <0.01, ^***^P <0.001

Results of western blot analysis showed that eNOS and iNOS levels in the lung tissues were comparable among the three treatment groups (Fig. 3a). However, eNOS activity, as assessed by ELISA, was significantly lower in the PP group compared with the PP-ABH and control groups (2.8 ± 1.3 U/L vs. 14 ± 5.4 U/L and 11 ± 3.5 U/L, respectively, *P* <0.01 and *P* <0.05, respectively) (Fig. 3b). Similarly, iNOS activity in the lung tissue was significantly higher in the PP group compared with the controls (19 ± 6.0 U/L vs 4.3 ± 1.9 U/L, *P* <0.001), but this increase was prevented in the PP-ABH group (11 ± 3.5 U/L).

### Oxidative stress

**Fig. 4:**
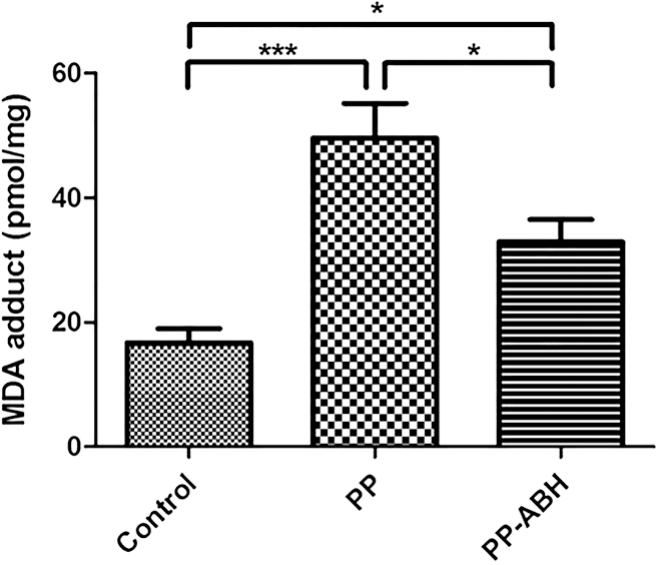
Oxidative stress. Levels of the oxidative stress marker malondialdehyde (MDA) were increased in the PP and PP-ABH groups compared to the control group, and were higher in the PP group than in the PP-ABH group. ^*^P <0.05, ^***^P <0.001

Results of ELISA showed that MDA content in lung tissues was higher in the PP and PP-ABH groups compared with the control group (50 ± 12 pmol/mg and 33 ± 8.1 pmol/mg vs. 17 ± 5.0 pmol/mg, respectively, *P* <0.001 and *P* <0.05, respectively), with higher MDA levels observed in the PP group than in the PP-ABH group (*P* <0.05) (Fig. 4).

### Inflammatory cytokines

**Fig. 5:**
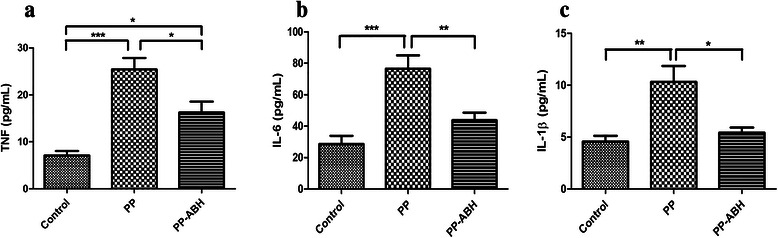
Inflammatory cytokines. **a** Serum levels of TNF-α were increased in the PP and the PP-ABH groups compared with the control group, and were higher in the PP group than in the PP-ABH group. **b** IL-6 and **c** IL-1β were increased significantly in the PP group compared with the PP-ABH and control groups. ^*^P <0.05, ^**^P <0.01, ^***^P <0.001

Results of ELISA showed that TNF-α expression was higher in the PP and PP-ABH groups compared with controls (25 ± 5.4 pg/mL and 16 ± 5.2 pg/mL vs. 7.1 ± 2.1 pg/mL, respectively, *P* <0.001 and *P* <0.05, respectively), with higher TNF-α levels observed in the PP group than in the PP-ABH group (*P* <0.05). Levels of both IL-6 and IL-1β were significantly higher in the PP group compared with the PP-ABH and control groups (Fig. 5).

### Lung injury

**Fig. 6:**
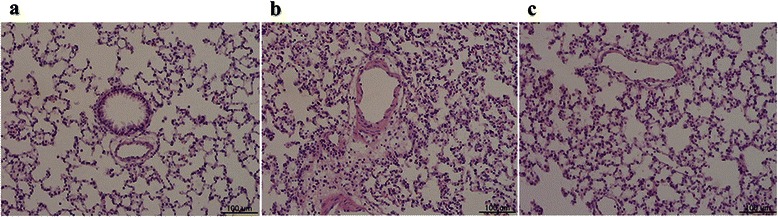
Lung histology. **a** Normal lung histology in the control group. **b** Lung tissue in the PP group shows inflammatory cell infiltration and protein exudation. **c** Pneumoperitoneum-induced inflammatory changes and protein exudation were attenuated in the PP-ABH group. Hemorrhage levels were comparable among the three groups

Results of histologic analysis showed increased cellularity in the PP group (*P* <0.001) and PP-ABH group (*P* <0.05) compared with controls, and cellular infiltration was greater in the PP group than in the PP-ABH group (*P* <0.001). Protein exudation was increased in the PP group compared the control (*P* <0.001) and PP-ABH groups (*P* < 0.01), but the degree of hemorrhage was similar among the three groups. The total lung injury scores (sum of cellularity, protein exudate, and hemorrhage scores) were 4.8-fold higher in the PP group (*P* <0.001) and 2-fold higher in the PP-ABH group (*P* <0.01) compared to the control group. The total lung injury score was significantly higher in the PP group than in the PP-ABH group (*P* <0.001) (Fig. 6, Table 1).

**Table 1:** Lung injury scores described by Kristof scoring system

	Cellularity	Protein exudation	Hemorrhage	Total score
Control	1.3 ± 0.58	0.66 ± 0.44	0.84 ± 0.36	2.9 ± 0.97
PP	7.4 ± 1.4^***^	4.8 ± 1.2^***^	1.4 ± 0.9	14 ± 1.4^***^
PP-ABH	3.4 ± 1.1^*, *****^	2.1 ± 0.93^****^	0.9 ± 0.43	6 ± 1.4^**, *****^

^*^P <0.05, ^**^P <0.01, ^***^P <0.001 compared with the control group

^****^P <0.01, ^*****^P <0.001 compared with the PP group

## Discussion

The main finding of this study was that administration of the arginase inhibitor ABH attenuated the pneumoperitoneum-induced decrease in NO bioavailability in rats. In addition, ABH maintained the activities of eNOS and iNOS; attenuated the increase in levels of the oxidative stress marker MDA and inflammatory cytokines TNF-α, IL-6, and IL-1β; and decreased the severity of lung injury caused by pneumoperitoneum.

Although pneumoperitoneum is essential for adequate visualization during laparoscopic surgery, the resulting derangement in organ function contributes to morbidity and mortality. The protective role of NO in maintaining organ function has been demonstrated in several studies. NO maintains the organ blood flow. Organ hypoperfusion during pneumoperitoneum can be attenuated by pretreatment with the NO substrates nitroglycerine and ethyl nitrite, but is exacerbated by administration of the NOS inhibitor NG-nitro-L-arginine methyl ester [6, 16]. In addition, NO protects the cardiovascular system by inhibiting norepinephrine secretion and decreasing the response of vascular smooth muscle to the sympathetic nervous system activity [12]. Furthermore, NO exerts anti-inflammatory and anti-thrombotic effects through the inhibition of leukocyte adhesion and platelet aggregation, thereby protecting against vascular diseases such as atherosclerosis [17, 18]. The predominant isoform of NOS is eNOS, which keeps blood vessels dilated, controls blood pressure, and has vasoprotective and anti-atherosclerotic effects [11]. In contrast, iNOS is induced by inflammatory cytokines, and NO produced by iNOS has cytostatic effects, contributing to the pathophysiology of inflammatory disease and septic shock [19]. In this study, pneumoperitoneum led to a decrease in eNOS activity and increase in iNOS activity, resulting in decreased NO bioavailability.

By competing with NOS for the substrate L-arginine, arginase acts as a critical regulator of NO synthesis [20]. Thus, arginase activity inhibits NO production [19], thereby decreasing NO levels and increasing superoxide, which can cause endothelial dysfunction [21]. Previous studies have reported that inhibition or knock-out of arginase decreases atherosclerosis and protects against myocardial ischemia-reperfusion injury [14, 22, 23]. Our results showed that NO bioavailability and eNOS activity were higher in rats that received ABH before pneumoperitoneum compared with those that did not receive ABH before pneumoperitoneum. It may suggest that inhibition of arginase activity could maintain NO bioavailability by preventing or attenuating pneumoperitoneum-induced changes in NOS activity.

It is well known that pneumoperitoneum-induced organ injury occurs not only in the intraabdominal organs, but also in the extraabdominal organ, such as lung. Pneumoperitoneum-induced oxidative stress and lipid peroxidation were identified as components of lung injury after pneumoperitoneum. The decrease of antioxidant renders cells or tissues more susceptible to reactive oxygen species attack [24] and lipid peroxidation results in the damage of biological membrane and therefore cell damage and tissue injury [25]. In addition, abdominal deflation at the end of a laparoscopic procedure provides a model of reperfusion damage in previously ischemic organs, aggravating the oxidative stress and lipid peroxidation [26]. Even when pneumoperitoneum was maintained at low pressure of 6 mmHg for 20 min, MDA and oxidatively modified proteins were increased in the lungs in rats [25]. With CO_2_ pneumoperitoneum maintained at 15 mmHg for 30 min, intra-alveolar hemorrhage, dense congestion, and leukocyte infiltration were found in the lungs in rats [27].

In addition, pneumoperitoneum increases the inflammation cytokine [9], which leads to the release of local mediators such as platelet-activating factors and reactive oxygen species, which cause damage to cell structures, capillary endothelium, and pulmonary tissue, resulting in lung injury [28]. Consistent with these previous findings, the levels of the oxidative stress marker MDA and inflammatory cytokines were increased by pneumoperitoneum in this study. Administration of a drug with anti-oxidant and anti-inflammatory effects was demonstrated to reduce the severity of pneumoperitoneum-associated lung injury [27, 29]. In this study, the pretreatment of arginase inhibitor before pneumoperitoneum partially or completely blocked the increase of oxidative stress and inflammation, and these anti-oxidant and anti-inflammatory effects might contribute to the decrease of lung injury.

As another possible mechanism to reduce lung injury, arginase inhibitor reduced iNOS activity. Oxidative stress is resulted from overproduction of reactive oxidative species, such as superoxide anion (O_2_^−^), which inactivates NO and diminishes its protective function [30]. Although NO protects against lung injury by inhibiting neutrophil migration and NF-κB activity [31, 32], excessive NO production by iNOS is associated with the development of lung injury in septic condition [33, 34]. In our results, the increased NO bioavailability and attenuated iNOS activity induced by the arginase inhibitor likely contribute to the reduction in lung injury.

This study has several limitations. Firstly, we decided the abdominal pressure at 15 mmHg, which was previously reported to induce oxidative injury in lung tissue in rats [8, 27]. In human, insufflation pressure is generally set at less than 12 mmHg, not to raise the intraabdominal pressure above that of the normal portal circulation pressure. Especially in surgical intensive care units, intra-abdominal pressure is tried to be maintained less than 12 mmHg to prevent abdominal compartment syndrome, where the detrimental effects of increased intra-abdominal pressure are frequently observed [35]. In this study, intraabdominal pressure was set at a higher level than usual to find out the effect of arginase inhibitor on pneumoperitoneum-induced organ injury. Although the majority of the studies investigating oxidative stress associated with pneumoperitoneum in rats have used the pressure of 12–15 mmHg, there is a possibility that these high pressures might exaggerate oxidative stress. In a rat model, pressures ≥ 8 mmHg are above the peritoneal capillary occlusion pressure and the optimal insufflation pressure to simulate human laparoscopy is known to approximate 5 mmHg [36]. In addition, the insufflation flow rate is also needed to be scaled down for rat size not to exaggerate oxidative stress. To apply our results to the clinical setting, further study is warranted in a rat model with physiologically relevant gas pressure and flow, which simulate the standard working conditions in humans. Secondly, although all study groups received mechanical ventilation with the same setting, elevated intraabdominal pressure might displace the diaphragm in a cephalad direction and increase intrathoracic pressure in the pneumoperitoneum-induced groups, which might influence lung injury. Thirdly, in addition to arginase and NOS, agmatine was reported to involve in the arginine-dependent pathways [37]. Agmatine is decarboxylated arginine, and thus agmatine biosynthesis competes with nitrogen metabolism by arginase and NO synthesis by NOS. Agmatine is induced in response to stress and/or inflammation and modulates NO synthesis by inhibition of neuronal NOS and iNOS and induction of eNOS. Further research is needed to elucidate the change of agmatine and the interaction among agmatine, NOS and arginase during pneumoperitoneum.

## Conclusions

In conclusion, our results show that pneumoperitoneum led to a decrease in eNOS activity and increase in iNOS activity, which might contribute to decreased NO bioavailability. Pretreatment with the arginase inhibitor, ABH, prevented these changes, and partially or completely blocked pneumoperitoneum-induced oxidative stress and inflammation, and decreased the severity of lung injury. Although these findings are not applicable to clinical practice, they indicate the potential of arginase inhibitors in reducing pneumoperitoneum-associated organ injury in laparoscopic surgery.

## Abbreviations

(NO): Nitric oxide
(NOS): NO synthase
(ABH): 2 (S)-amino-6-boronohexanoic acid
(eNOS): Endothelial NO synthase
(CO_2_): Carbon dioxide
(iNOS): Inducible NOS
(ELISA): Enzyme-linked immunosorbent assay
(MDA): Malonyldialdehyde
(TNF): Tumor necrosis factor
(IL): Interleukin
