# Erratum to: The effects of arginase inhibitor on lung oxidative stress and inflammation caused by pneumoperitoneum in rats

**DOI:** 10.1186/s12871-016-0288-9

**Published:** 2016-12-13

**Authors:** Jin Sun Cho, Young Jun Oh, Ok Soo Kim, Sungwon Na

**Affiliations:** 1 Department of Anesthesiology and Pain Medicine, Yonsei University College of Medicine, Seoul, Republic of Korea; 2 Department of Anesthesiology and Pain Medicine, Anesthesia and Pain Research Institute, Yonsei University College of Medic, Seoul, Republic of Korea

## Erratum

In this version of this article that was originally published [1] there is an error in the Acknowledgements. Where it says ‘This study was supported by a faculty research grant of Yonsei University College of Medicine (6-2010-0163)’, the number should be 6-2012-0012, not 6-2010-0163.
